# Apigenin inhibits renal cell carcinoma cell proliferation

**DOI:** 10.18632/oncotarget.15771

**Published:** 2017-02-28

**Authors:** Shuai Meng, Yi Zhu, Jiang-Feng Li, Xiao Wang, Zhen Liang, Shi-Qi Li, Xin Xu, Hong Chen, Ben Liu, Xiang-Yi Zheng, Li-Ping Xie

**Affiliations:** ^1^ Department of Urology, The First Affiliated Hospital, School of Medicine, Zhejiang University, Hangzhou, Zhejiang Province 310003, China

**Keywords:** renal cell carcinoma, apigenin, DNA damage, ATM signaling, apoptosis

## Abstract

Apigenin, a natural flavonoid found in vegetables and fruits, has antitumor activity in several cancer types. The present study evaluated the effects and mechanism of action of apigenin in renal cell carcinoma (RCC) cells. We found that apigenin suppressed ACHN, 786-0, and Caki-1 RCC cell proliferation in a dose- and time-dependent manner. A comet assay suggested that apigenin caused DNA damage in ACHN cells, especially at higher doses, and induced G2/M phase cell cycle arrest through ATM signal modulation. Small interfering RNA (siRNA)-mediated p53 knockdown showed that apigenin-induced apoptosis was likely p53 dependent. Apigenin anti-proliferative effects were confirmed in an ACHN cell xenograft mouse model. Apigenin treatment reduced tumor growth and volume *in vivo*, and immunohistochemical staining revealed lower Ki-67 indices in tumors derived from apigenin-treated mice. These findings suggest that apigenin exposure induces DNA damage, G2/M phase cell cycle arrest, p53 accumulation and apoptosis, which collectively suppress ACHN RCC cell proliferation *in vitro* and *in vivo*. Given its antitumor effects and low *in vivo* toxicity, apigenin is a highly promising agent for treatment of RCC.

## INTRODUCTION

As a natural phytoestrogen flavonoid widely distributed in vegetables and fruits [1], apigenin (4′,5,7-trihydroxyflavone) effectively inhibits proliferation in multiple cancer cell types, including colon, lung, breast, prostate, melanoma, and leukemia [2–7] and DNA damage mechanisms were comprised in lots of studies [8]. Apigenin may also exhibit reduced systemic toxicity compared to other flavonoids [9]. Despite reported apigenin sensitivity in several cancer types, few studies have assessed apigenin in renal cancer cells.

Renal cell carcinoma (RCC) is the most common type of adult malignant kidney tumor. RCC is also the most lethal of the common urological cancers; despite diagnostic advances, 25–30% of patients present with metastasis. Metastatic RCC patient prognosis is poor, and median survival can be less than one year [10]. Because RCC is frequently resistant to chemo- and radiotherapy, surgical resection remains the main treatment modality, especially for organ-confined disease [11]. Suitable therapeutic agents must not only reduce RCC proliferation, but should also have low toxicity in healthy tissues. We designed the present study to investigate whether apigenin had anti-proliferation activity in RCC cell lines *in vitro* and *in vivo*. We observed for the first time that apigenin exposure induced DNA damage, G2/M phase cell cycle arrest, and apoptosis in ACHN RCC cells via ATM serine/threonine kinase signaling modulation and the p53 pathway.

## RESULTS

### Apigenin inhibits ACHN, 786-0, and Caki-1 cell proliferation *in vitro*

**Figure 1:**
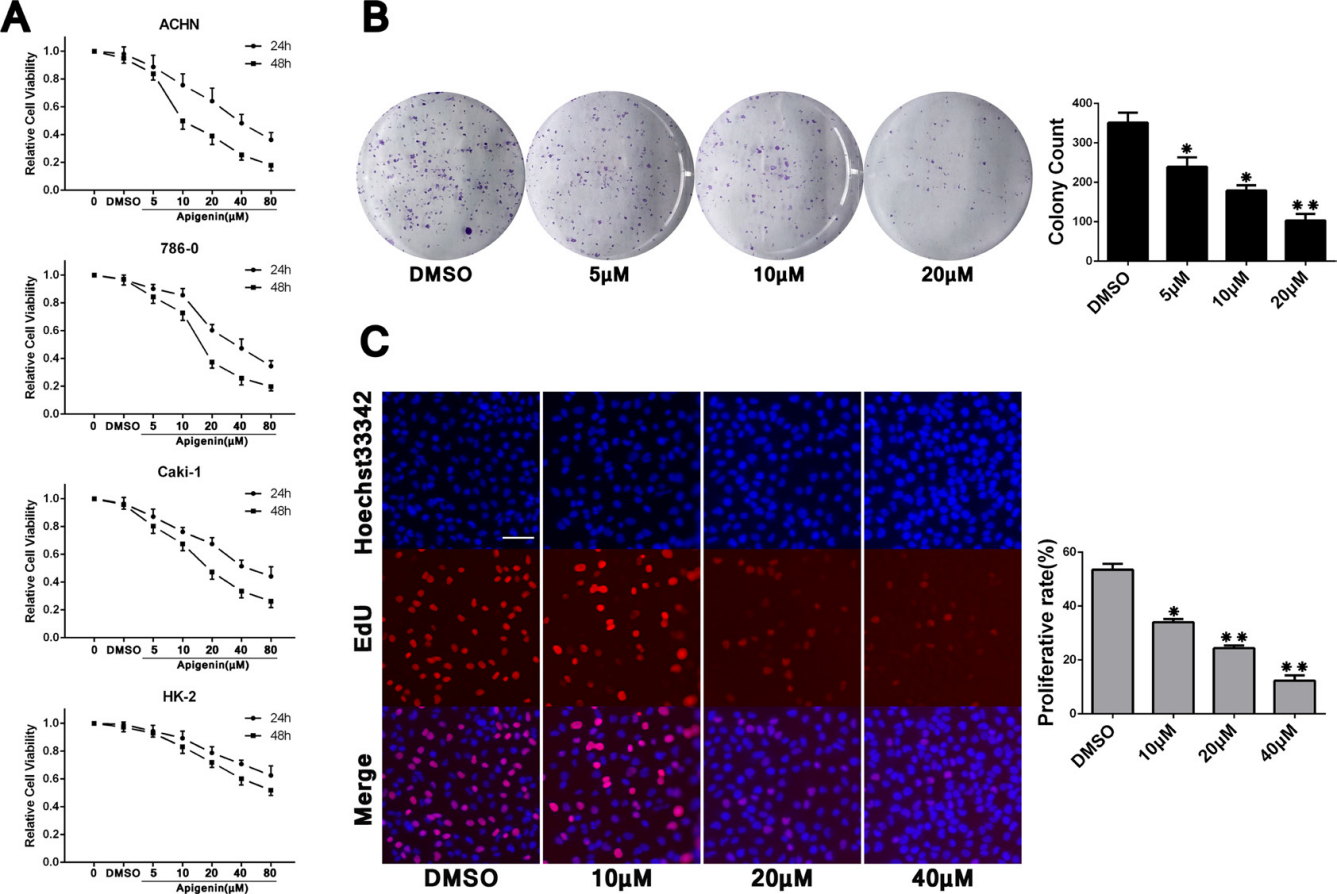
Apigenin inhibits ACHN, 786-0, and Caki-1 cell proliferation CCK-8 analysis showed apigenin inhibited proliferation in ACHN, 786-0, and Caki-1 RCC cells, and the normal renal proximal tubule epithelial cell line, HK-2, in a dose- and time-dependent manner (**A**) This analysis was repeated three times, with six replicates per experiment. Representative colony formation assay images and quantitation (**B**). Cell counts > 50 were defined as a colony. Representative EdU proliferation assay images using a 10× objective (**C**) ACHN cell proliferation rates were calculated as the ratios of EdU-staining cells to Hoechst33342-staining cells from six fields of three separate experiments. Scale bar = 25 μm. *P < 0.05, **P < 0.01 versus untreated control.

Cell Counting Kit-8 (CCK-8) analysis indicated that treatment with apigenin at varying concentrations (5–80 μM) inhibited proliferation of ACHN, 786-0, and Caki-1 RCC cells, and of the normal renal proximal tubule epithelial cell line, HK-2, in a dose- and time-dependent manner (Figure 1A). IC50s for each cell line were: ACHN (39.4 μM for 24 h; 15.4 μM for 48 h), 786-0 (39.1 μM for 24 h; 19.0 μM for 48 h), Caki-1 (50.9 μM for 24 h; 21.4 μM for 48 h) and HK-2 (122.1 μM for 24 h; 70.2 μM for 48 h). HK-2 cells were less susceptible to apigenin than RCC cells. Apigenin sensitivity was similar in the three RCC cell lines, and we paied close attention to one of them (ACHN) for our mechanistic studies. Colony formation and EdU proliferation assays were undertaken to assess apigenin-induced proliferation changes. Compared to the vehicle control (DMSO, 1 μl/ml), ACHN cells treated with different concentrations of apigenin formed fewer colonies (Figure 1B) and incorporated less EdU (Figure 1C). These results show reduced proliferation rates in apigenin-treated RCC cells compared to controls.

### Apigenin causes DNA damage in ACHN cells

**Figure 2:**
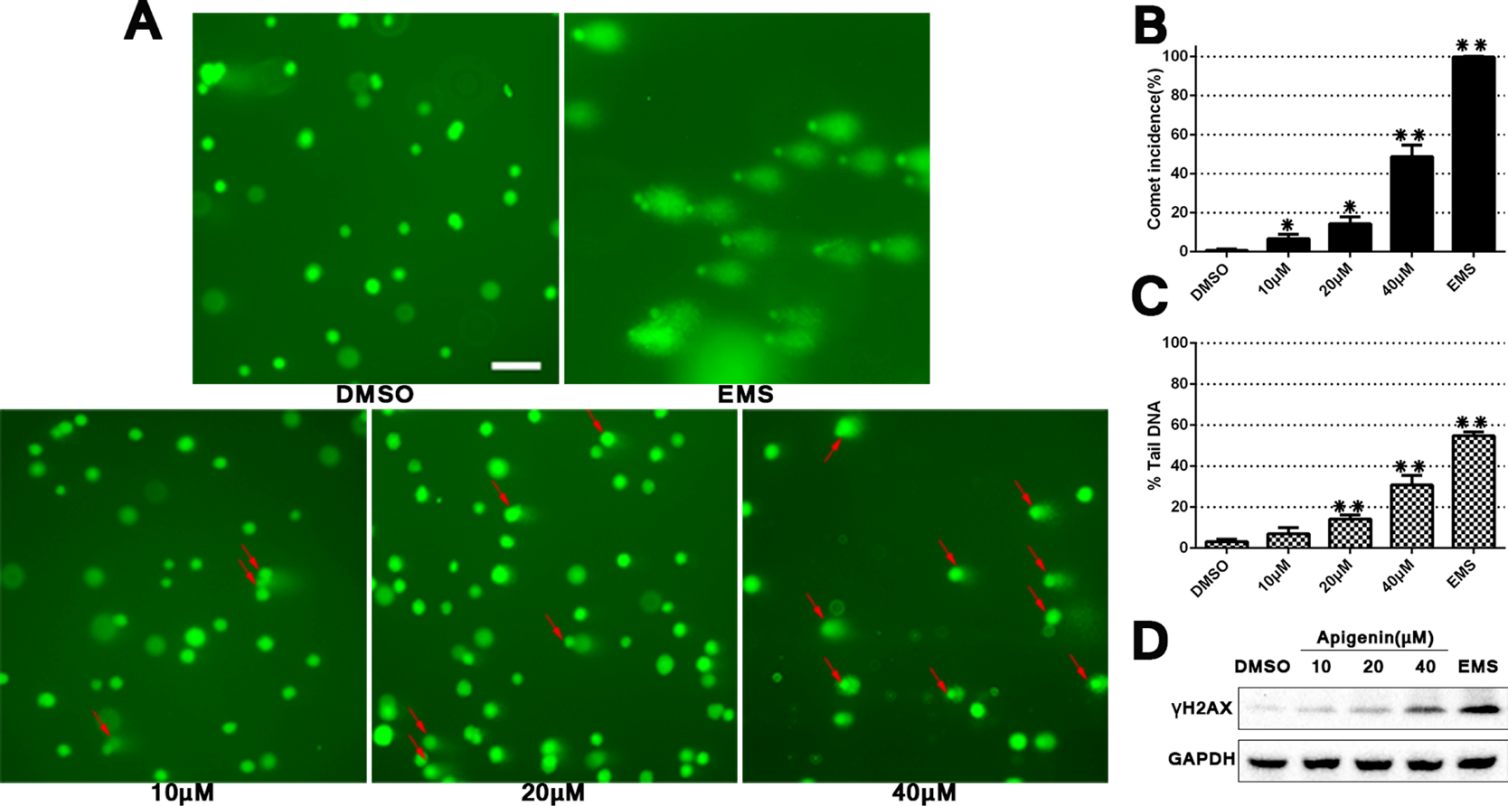
Apigenin treatment induces DNA damage in ACHN cells Comet assay using 4S Green Plus dye following cell treatment with various apigenin concentrations (**A**) Red arrows specify cells with a “comet tail,” which indicates DNA damage. Scale bar = 25 μm. Comet formation incidences in each group were measured using 100 randomly selected cells, and DNA damage level was calculated using CaspLab software in accordance with % tail DNA (**B**–**C)**. Western blotting analysis of γH2AX D. *P < 0.05, **P < 0.01 versus untreated control.

Alkaline single cell gel electrophoresis (a comet assay) was conducted to determine whether apigenin induced DNA damage in ACHN cells. A known DNA damaging agent (EMS, ethyl methanesulfonate) was included as a positive control. Comet formation incidence and DNA damage degree were quantified (Figure 2). DNA damage degree was evaluated by comparing comet “head” and “tail” fluorescence intensity using CaspLab software. Apigenin exposure induced DNA damage and increased comet formation in a dose-dependent manner compared to DMSO controls. Additionally, western blotting showed a progressive increase in phosphorylated H2AX (γH2AX), which is a marker for double-stranded DNA breaks. Collectively, these data implied that apigenin treatment induces DNA damage in ACHN cells.

### Apigenin triggers G2/M phase cell cycle arrest and stimulates cell cycle factors through ATM signaling

**Figure 3:**
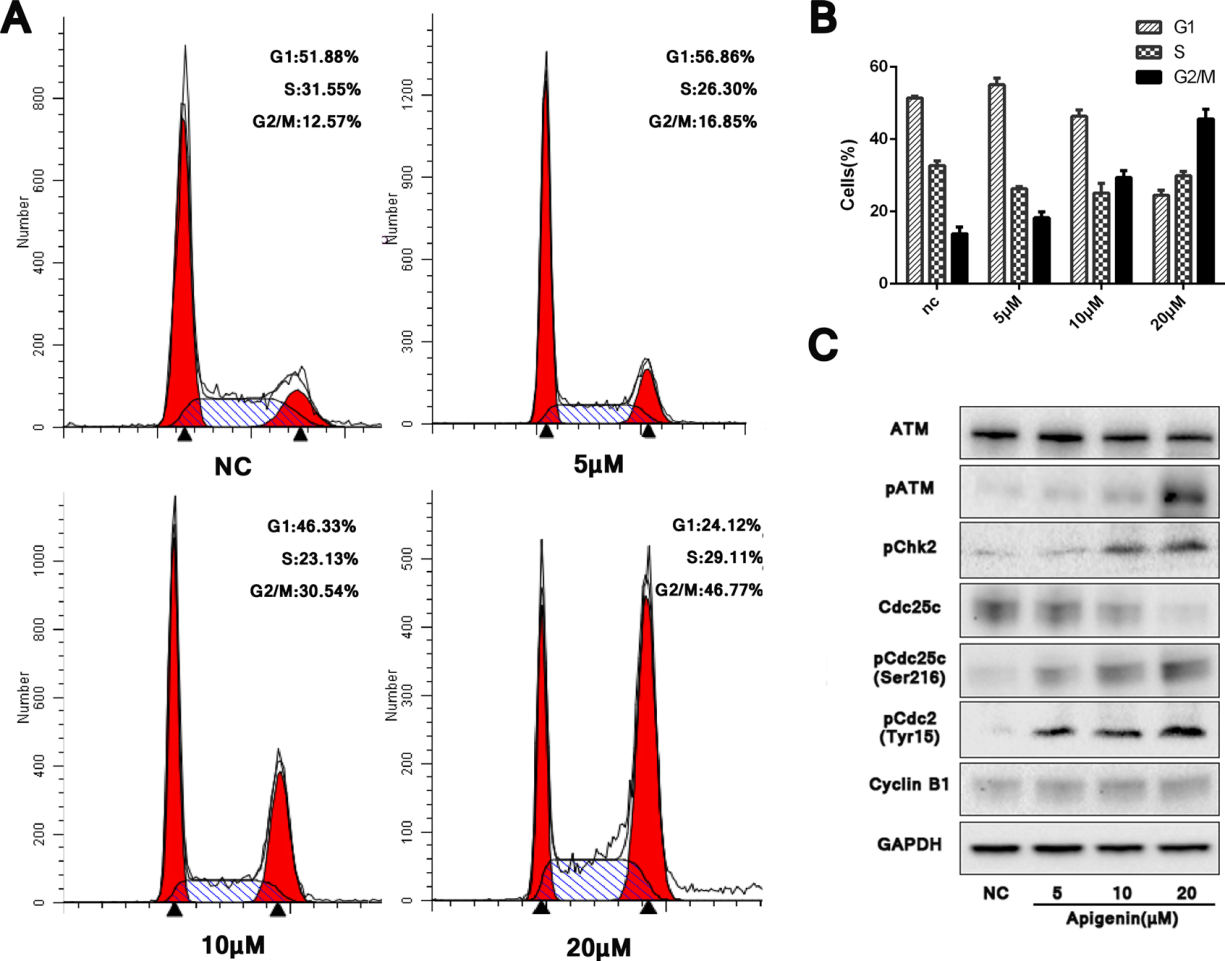
Apigenin causes G2/M phase cell cycle arrest and modulates cell cycle factors through ATM signaling Apigenin induced G2/M phase cell cycle arrest in a dose-dependent manner (**A**–**B)**. Western blotting analysis of ATM pathway proteins (**C**). *P < 0.05, **P < 0.01 versus untreated control.

Appreciable cell cycle arrest in G2/M phase was observed in apigenin treated ACHN cells compared with untreated controls. Cell cycle analyses from three independent experiments showed that in treated cells, the G2/M phase-arrested population increased to 16.85%, 30.54% and 46.77% with 5, 10 and 20 μM apigenin, respectively, up from 12.57% in controls (Figure 3A–3B). Treatment also reduced the G1 phase population.

ATM/ATR signaling reportedly plays a functional role in DNA damage-induced G2/M phase arrest. Western blotting for ATM and p-ATM after treatment with varying concentrations of apigenin for 48 h showed that p-ATM increased dose-dependently, despite no obvious difference in total ATM (Figure 3C). Phosphorylated Chk2 (p-Chk2), the major downstream effector activated by ATM, as well as phosphorylated Cdc25c (p-Cdc25c on serine 216) and phosphorylated Cdc2 (p-Cdc2 on tyrosine15), also increased. Successful transition through G2/M phase requires dephosphorylation of Cdc2 and Cyclin B1. Hardly difference was found on the expression level of Cyclin B1, but increased pCdc2 tended to reduce the formation of dephosphorylated Cdc2 and Cyclin B1 compound, therefore induced the final G2/M phase arrest. Our results confirmed DNA damage-induced G2/M phase arrest following apigenin treatment, which activated ATM-mediated cell cycle checkpoint signaling.

### Apigenin induces apoptosis in ACHN cells

**Figure 4:**
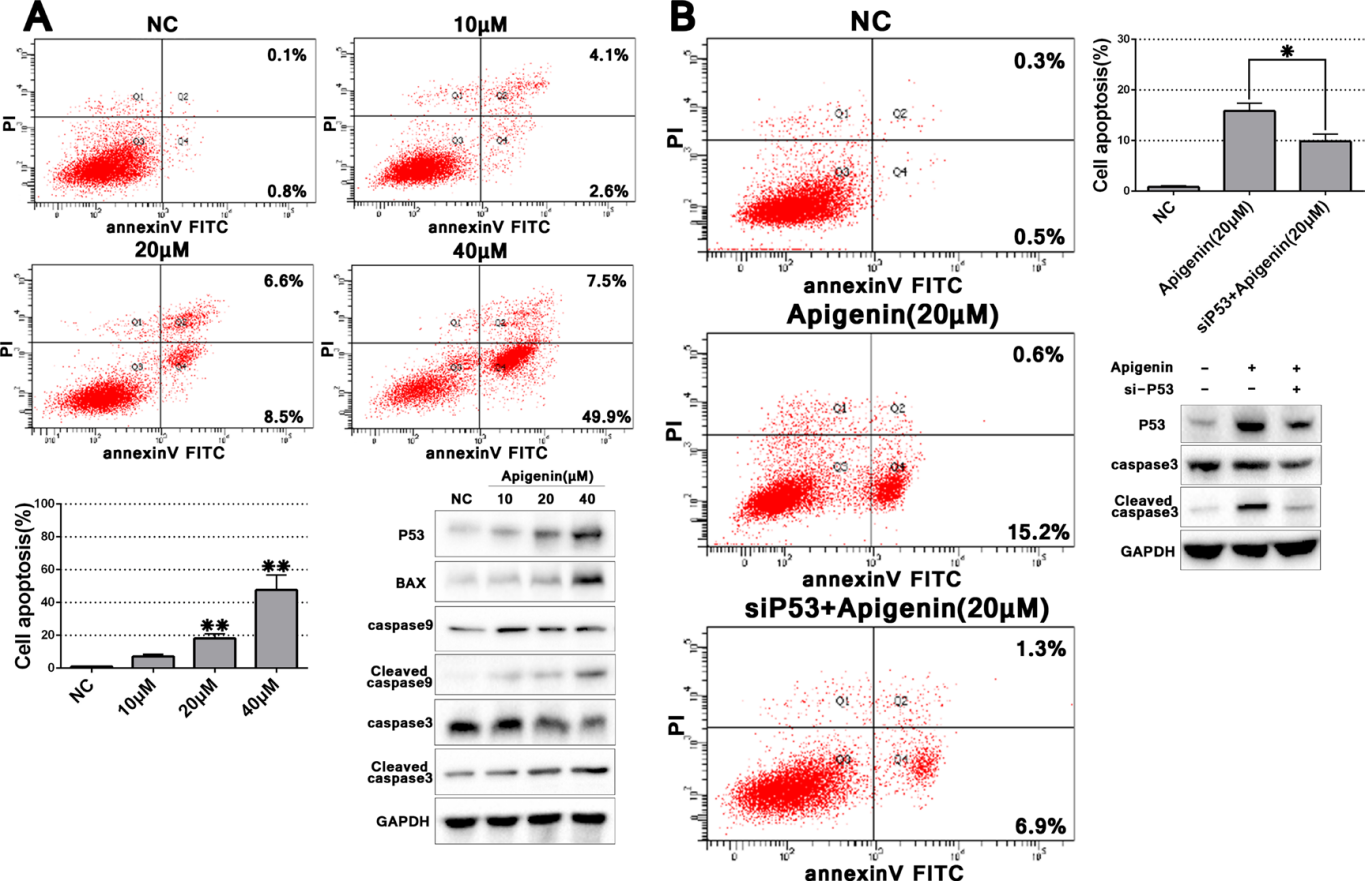
Apigenin induces ACHN cell apoptosis, and p53 knockdown partially rescues this effect Representative flow cytometry apoptosis assay images and quantitation of one experiment done in triplicate (**A**) p53 knockdown partially rescued apigenin-induced apoptosis (**B)** *P < 0.05, **P < 0.01.

We observed enhanced ACHN cell apoptosis at higher apigenin concentrations (> 20 μM) compared with controls (Figure 4A). Total apoptosis rates following treatment with 20 and 40 μM apigenin were 15.1 and 57.4%, respectively. To assess whether genotoxicity was responsible for increased apoptosis at higher apigenin doses, we evaluated p53 expression with varying concentrations of apigenin (10, 20, and 40 μM). Because p53 increased dose-dependently after apigenin treatment, we analyzed several key effectors downstream of p53-induced apoptosis signaling. Expression of the pro-apoptotic protein BAX, and cleaved caspases 9 and 3 increased after apigenin treatment, suggesting that apigenin-induced apoptosis may depend on p53 signaling.

### p53 knockdown partially rescues apigenin-induced apoptosis

To further examine the role of p53 signaling in apigenin-induced apoptosis, we knocked down p53 via specific siRNA (sip53). Apigenin treatment (20 μM)-induced p53 upregulation was rescued by sip53, but not by the scrambled sip53 control or negative control (siNC) (Supplementary Figure 1 and Figure 4B). Cleaved caspase 3 was also reduced in sip53-transfected cells compared to controls. Cell apoptosis as assessed via flow cytometry also decreased in sip53-transfected and apigenin-treated cells as compared to cells treated with apigenin alone. These results suggested that apigenin-triggered apoptosis is partially rescued by p53 knockdown.

### Apigenin inhibits tumorigenicity *in vivo*

**Figure 5:**
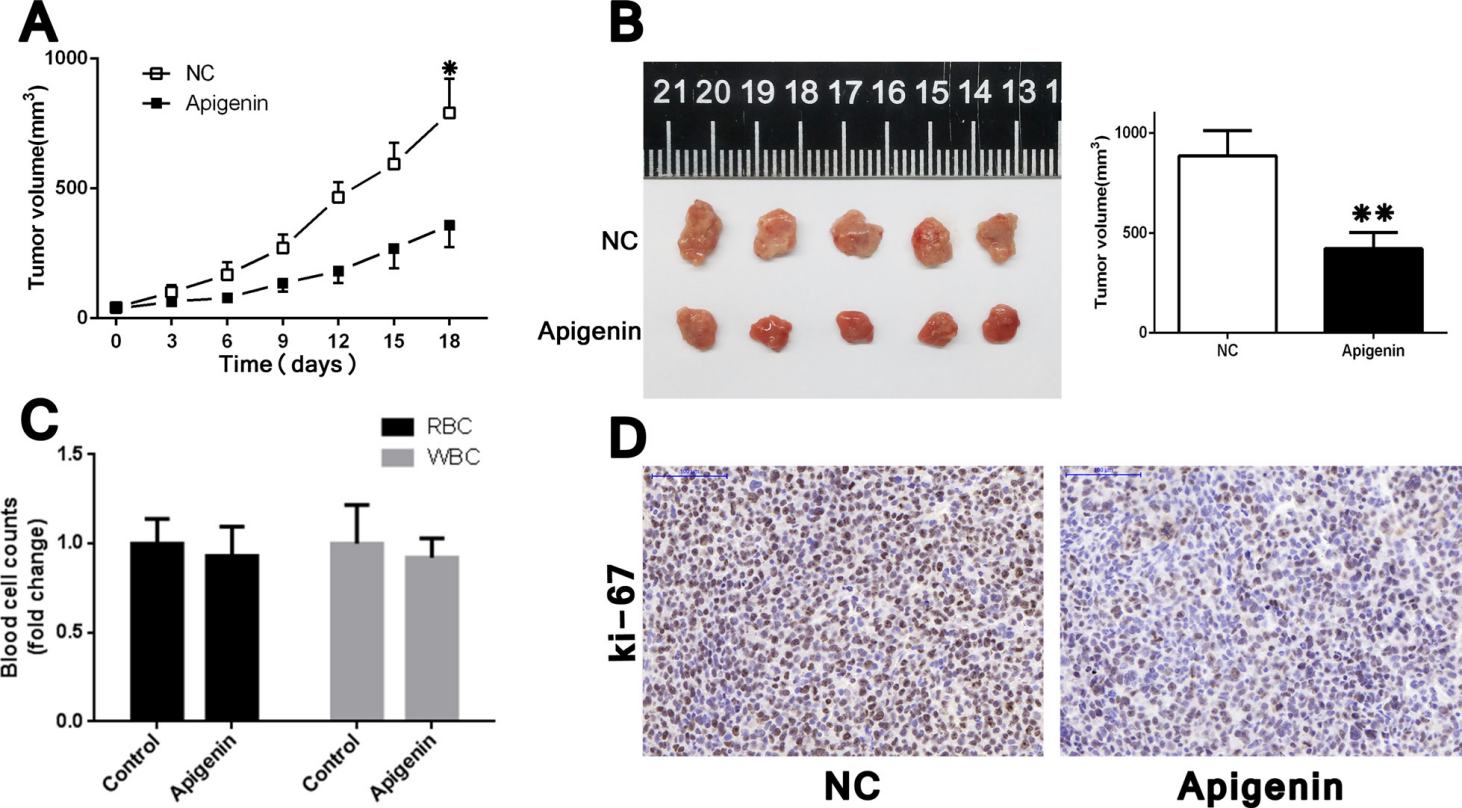
Apigenin inhibits tumorigenicity in ACHN cell xenograft nude mice Tumor xenograft model (**A**) ACHN cells (1 × 10^6^ in 100 μl PBS) were injected subcutaneously into the right flank of each mouse. After palpable tumors arose, apigenin (or vehicle alone) was administered at 30 mg/kg every 3 d intraperitoneally. Tumors grew more slowly in apigenin-treated mice. Harvested tumors represented smaller ones in apigenin-treated group (**B)**. Apigenin treatment did not affect end point RBC and WBC counts (**C**). Apigenin treated tumors had reduced Ki-67 expression (**D**). Scale bar = 100 μm. Error bars represent the S.D. from five nude mice. *P < 0.05, **P < 0.01.

We examined the effects of apigenin treatment *in vivo* using a xenografted nude mouse model, in which ACHN cells were subcutaneously injected into the right flank of each animal. No mice died as a result of apigenin toxicity. RBC (red blood cell) and WBC (white blood cell) counts at the treatment end point (21 d) were the same between apigenin-treated mice and controls (Figure 5C). Apigenin treatment reduced tumor growth and volume (Figure 5A–5B), and IHC staining revealed lower Ki-67 indices in tumors derived from apigenin-treated mice (Figure 5D).

## DISCUSSION

Since the 1940s, botanical extracts and their derivatives have been applied as small molecule anti-tumor agents [12]. As a plant-produced compound, apigenin is found in many foods, including parsley, oranges, teas, chamomile, onions, wheat sprouts, and others [1, 13]. Compared to other flavonoids, apigenin's low general toxicity supports its potential use as an anticancer agent [9]. Other reported apigenin biological activities include anti-oxidation [14], anti-inflammation [15, 16], UVB-induced mutation stabilization [17], anti-virus [18], and others. These activities rely on a multitude of molecular mechanisms, such as cell cycle arrest, apoptosis, stress attenuation, and immune system modulation [18–21].

Our results suggest for the first time that apigenin exposure causes DNA damage in ACHN RCC cells, which may trigger DNA damage repair systems, and finally induce G2/M cell cycle arrest and apoptosis. Sharma, *et al*. [22] reported that apigenin directly bound DNA in prostate epithelial cell lines, although the mechanisms by which this binding may induce DNA damage must be further explored. Apigenin-induced G2/M phase accumulation and apoptosis have been frequently reported [4, 5, 23–25]. However, delayed cell cycle progression at G1/S phase is also observed in several kinds of malignant tumors [26–28]. Apigenin's impacts on cell cycle progression must be addressed in various types of cancers.

Multiple signaling pathways are reportedly modulated by apigenin treatment. In liver cancer cells, apigenin is a kinase antagonist and inhibits the ERK (peroxisome proliferation regulated kinase) pathway [29]. Shukla, *et al*. suggested apigenin suppressed IKKα and downstream NF-κB signaling molecules, repressing prostate cancer progression [30]. Apigenin induces apoptosis in human anaplastic thyroid cancer cells through downregulation of both EGFR tyrosine autophosphorylation and phosphorylation of its downstream effector, mitogen activated protein (MAP) kinase [31]. We found here that apigenin treatment activated ATM signaling, triggering p-Cdc2 accumulation, and inducing G2/M phase arrest. ATM phosphorylates p53 directly or indirectly through Chk2, thus modulating cell cycle progression and apoptosis. This interaction could explain apigenin-induced p53 accumulation. Additionally, increased p53 mRNA was also detected after apigenin treatment and partial rescue of apigenin-induced apoptosis by sip53 further supports a key role for p53 in this process.

However, p53 regulation-independent apigenin-induced apoptosis has also been reported. In p53-mutant cell lines, HT-29 and MG63, a luciferase reporter assay using a p53-responsive promoter plasmid was not activated by apigenin treatment [32]. Together with our results, this implies that apigenin could be effective not only in wild-type p53 status tumors, but also in cancers with mutant p53. Banerjee, *et al*. observed that apigenin-induced oxidative stress led to colorectal cancer cell senescence, possibly through p53-independent signaling mechanisms [33]. Additionally, mitochondria-mediated apoptosis was demonstrated in several apigenin-treated cancer cell types [33–35]. Kobayashi, *et al*. tested multiple flavonoids in human LNCaP prostate cancer cells, and suggested that apigenin induced p53-dependent p21 production, but luteolin did so independent of p53 [36]. This discrepancy may depend on cancer cell p53 status (mutant or wild type, and expression level differences), or the status of p53 modulators following apigenin treatment. Nonetheless, our study showed that in ACHN RCC cells, DNA toxicity-triggered ATM phosphorylation leads to p53 accumulation, followed by apoptosis.

Given RCC insensitivity to various chemotherapeutics, radiotherapy, and non-specific immunotherapies, the present first line treatment for patients with favorable- or intermediate-risk clear cell RCC (ccRCC) is targeted therapy. This includes vascular endothelial growth factor antibody (VEGF-AB), VEGF receptor tyrosine kinase inhibitors (TKI), and mammalian target of rapamycin (mTOR) inhibitors, namely sunitinib, pazopanib, temsirolimus, and others [37]. Apigenin not only indirectly inhibits mTOR, Raf, and VEGF expression [17, 38, 39], but also may directly bind and antagonize VEGFR, similar to TKI [40]. Apigenin appears to be a highly promising agent for RCC treatment, likely through various biological mechanisms.

In conclusion, apigenin induces G2/M phase arrest and apoptosis in ACHN RCC cells, possibly through direct DNA damage. Apigenin treatment activated ATM-mediated cell cycle checkpoint signaling, and led to p53 accumulation. Xenografted mice treated with apigenin exhibited reduced tumor growth rates and volumes. Given its antitumor effects and low *in vivo* toxicity, apigenin is a highly promising agent for treatment of RCC.

## MATERIALS AND METHODS

### Reagents and cell culture

Apigenin was obtained from Sigma (St. Louis, MO, USA) and dissolved in DMSO (vehicle). Final DMSO concentrations used *in vitro* were ≤ 0.2%. Antibodies specific to GAPDH, p53, BAX, Cyclin B1, caspase 3 and caspase 9 were purchased from Santa-Cruz Biotechnology (Santa Cruz, CA, USA). ATM, p-ATM, γH2AX, p-Chk2, Cdc25c, p-Cdc25c (Ser216) and p-Cdc2 (Tyr15) antibodies were purchased from CST (Danvers, MA, USA) and applied according to the manufacturer's protocol.

Human RCC cell lines, ACHN, 786-0, and Caki-1, were obtained from the Shanghai Institute of Cell Biology, Chinese Academy of Sciences (Shanghai, China) and cultured in MEM, RPMI 1640, and McCOY's 5A medium (HyClone, Logan, UT, USA), respectively, supplemented with 10% heat-inactivated fetal bovine serum at 37°C in 5% CO_2_.

### Cell proliferation assay

Approximately 4 × 10^3^ cells per well were plated in 96-well plates. After incubation overnight, cells were treated with different concentrations of apigenin dissolved in DMSO for 24 or 48 h. At different time points, medium was removed and cell-counting solution (WST-8, Dojindo Laboratories, Kumamoto, Japan) was added to each well. Following incubation at 37°C for 1 h, absorbance was measured spectrophotometrically at 450 nm with a MRX II microplate reader (Dynex Technologies, Chantilly, VA, USA). IC50 was calculated using Calcusyn software (Biosoft, Cambridge, UK).

### Colony formation assay

Following apigenin treatment for 24 h, cells were rinsed, trypsinized, resuspended as single cells, and plated in six-well culture plates at 800 cells per well for approximately two weeks. Cells were then fixed with methanol and stained with 0.1% crystal violet. Colony numbers and mean cell numbers for each colony were counted under an optical microscope (Olympus, Shanghai, China).

### EdU incorporation assay

We assessed cell proliferation by detecting EdU (5′-ethynyl-2′-deoxyuridine) incorporation. EdU is a nucleotide analog of thymidine, and is incorporated into DNA only in proliferating cells. Following incubation with EdU for 2 hours, a fluorescent molecule from the kit that reacts specifically with EdU was added to visualize proliferating cells using a fluorescence microscope (Olympus). Using the EdU DNA Cell Proliferation Kit (Guangzhou Ribobio, Guangzhou, China) in accordance with the manufacturer's instruction, proliferating cell frequencies were calculated as the ratios of EdU-staining cells to Hoechst33342-staining cells counted from six fields of three separate experiments.

### Genotoxic effect of apigenin evaluated via comet assay

To clarify the genotoxic effect of apigenin on ACHN cells, we carried out a comet assay, which was performed as described by Tice, *et al*., except with the DNA dye replaced with 4S Green Plus (Sangon, Shanghai, China). All slides were observed under fluorescence microscope (Olympus). Cells treated with a known DNA damaging agent (4 mM EMS, ethyl methanesulfonate) for 2 h were included as a positive control. Comet formation incidence rates in each group were measured using 100 randomly selected cells. DNA damage level was calculated using the Comet Assay Software Project (CaspLab) in accordance with % tail DNA.

### Cell cycle analysis via flow cytometry

Approximately 9 × 10^4^ cells per well were seeded in six-well plates, incubated overnight, and treated with different concentrations of apigenin. Cells were washed twice with PBS 48 h later, then collected and fixed in 75% ethanol overnight at 4°C. Cells were then washed twice with PBS, resuspended in propidium iodide (PI) solution (50 μg/ml) containing RNase A, and incubated for 30 min at room temperature. The BD LSRII Flow Cytometer System with FACSDiva Software (BD Bioscience, Franklin Lakes, USA) was used for cell cycle analysis. Data were analyzed using ModFit LT 3.2 software (Verity Software House, Topsham, USA).

### Cell apoptosis analysis via flow cytometry

Cells were harvested from six-well plates and washed with PBS 48 h after apigenin treatment. Cells were then incubated for 15 min in mixed binding buffer (containing annexin V-FITC and PI) in the dark, according to the manufacturer's instructions. Analysis was carried out using the BD LSRII Flow Cytometer System with FACSDiva Software (BD Bioscience, Franklin Lakes, USA) within 1 h. FACSDiva software was also used for quantifying the percentage of apoptotic cells.

### Western blotting analysis

Following apigenin treatment, cells were rinsed, collected, lysed with lysis buffer (5 mmol/L EDTA, 10 mmol/L Tris-HCl, 0.25 mol/L sucrose, 50 mmol/L NaCl, 30 mmol/L sodium pyrophosphate, 50 mmol/L NaF, 1 mmol/L Na_3_VO_4_, 1 mmol/L PMSF, and 2% cocktail [pH 7.5]), and quantified using a BCA kit (Thermo, USA). Proteins (20–30 μg) were separated via 10% SDS-PAGE and transferred to PVDF membranes. Membranes were blocked with 5% non-fat milk and then incubated overnight with antibodies at dilutions specified by the manufactures. After three rinses in TBST on a shaking table, membranes were incubated in horseradish peroxidase-conjugated goat anti-rabbit secondary antibody for 1 h. Membranes were then washed in TBST three times, detected using an enhanced chemiluminescence (ECL) system (Pierce, Biotechnology Inc., Rockford, USA), and analyzed via the Bio-Rad ChemiDoc MP system (Bio-Rad, CA, USA).

### siRNA and transfection

The siRNA targeting human TP53 mRNA, the negative control with no significant homology to any known human sequences, the fluorescein-labeled siRNA used for affirming transfection efficiency, the positive control (siGAPDH), and scrambled siRNA control were purchased from GenePharma (Shanghai, China) (Supplementary Table 1). Transfections were performed using Lipofectamine 2000 reagents (Invitrogen, Carlsbad, CA, USA) according to the manufacturer's protocol 8 h before treatment with apigenin or DMSO control. Transfection efficiency was assessed using real-time PCR and western blotting for the corresponding genes.

### Reverse transcription–PCR and real-time PCR

Total RNA was isolated with TRIzol reagent (Takara, China) and reverse transcribed into cDNA using the PrimeScript RT reagent Kit (Takara, China). Real-time PCR quantification with SYBR Green (Takara, China) was performed using an ABI 7500 fast real-time PCR System (Applied Biosystems, USA) with 96-well plates in 10 μl total volume per reaction. Relative GAPDH and TP53 levels normalized by ACTB mRNA were analyzed using the 2^−ΔΔCt^ method. All qPCR primers were provided by Sango Biotech (China) (Supplementary Table 1).

### *In vivo* tumorigenicity assay

Male BALB/c-nude mice (4 weeks old) were purchased from the Shanghai Experimental Animal Center, Chinese Academy of Sciences, Shanghai, China. All mice were kept in a pathogen-free environment and fed ad lib. Animal care and use procedures were approved by the Ethics Committee of the First Affiliated Hospital of the Medical College, Zhejiang University (Hangzhou, P.R. China), and all applicable institutional and national rules concerning the ethical use of animals were followed. ACHN cells (1 × 10^6^ in 100 μl PBS) were injected subcutaneously into the right flank of each mouse. After palpable tumors arose (the average tumor sizes are 41.66 mm^3^ for NC and 36.6 mm^3^ for apigenin treatment group), apigenin (or vehicle control) was administered at 30 mg/kg every 3 d intraperitoneally for 21 d. Tumor size was measured with a caliper before each apigenin injection and calculated using the formula V = (width^2^ × length × 0.52). Blood samples were collected from the tail vein at 21 d for RBC and WBC counts using a cell counting chamber.

### Immunohistochemistry

Immunohistochemistry (IHC) staining was performed on tumors harvested from nude mice using an anti-Ki-67 antibody. Briefly, tumor tissues were fixed, paraffinized, and sectioned. Following dewaxing and rehydration, sections were heated in sodium citrate buffer to retrieve antigens. Then slides were blocked in BSA (Sango Biotech, Shanghai, China), incubated with anti-Ki-67 antibody (Epitomics, Burlingame, USA) overnight at 4°C, and then incubated with HRP-conjugated goat anti-rabbit secondary antibody for 1 h at room temperature. DAB was used for color development, so dark brown was regarded as positive. Positivity strength was semi-quantified by considering both staining intensity and the proportion of positive cells.

### Statistical analysis

Data shown figures are expressed as means ± SD from three independent experiments performed separately, unless stated otherwise. All analyses were developed using GraphPad Prism v5 for Windows. Differences between treatment groups and controls were estimated using the χ^2^-test or Student's *t*-test. A two-tailed value of *P* < 0.05 was considered statistically significant.

## SUPPLEMENTARY MATERIALS FIGURE AND TABLE


