# Supplementary material for: Apigenin inhibits renal cell carcinoma cell proliferation
Source: Oncotarget. 2017 Feb 28;8(12):19834–42. doi: 10.18632/oncotarget.15771 (PMC5386726; doi:10.18632/oncotarget.15771)
Supplement: Supplementary file 1 [file oncotarget-08-19834-s001.pdf]

Apigenin inhibits renal cell carcinoma cell proliferation

Supplementary Materials

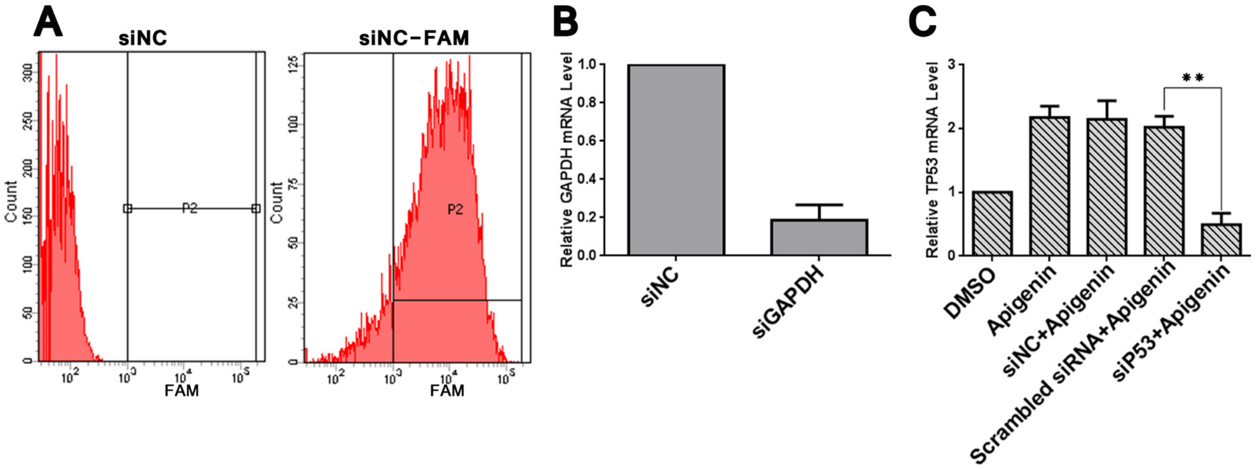

Supplemental Figure 1: siRNA transfection efficiency and p53 mRNA level in ACHN cells. Fluorescein-labeled siRNA had a higher transfection efficiency than non-fluorescent siNC (**A**) siGAPDH was used as a positive control (**B**) p53 mRNA increased after apigenin treatment (20 µM) and decreased following sip53 transfection, but not scrambled sip53 or siNC transfection (**C**) $^{**}P < 0.01$.

Supplementary Table 1: The oligonucleotides used in this study

Name[a]	Sequence(5′ → 3′)
ACTB F	CACCATTGGCAATGAGCGGTTC
ACTB R	AGGTCTTTGCGGATGTCCACGT
GAPDH F	GTCTCCTCTGACTTCAACAGCG
GAPDH R	ACCACCCTGTTGCTGTAGCCAA
TP53 F	CCTCAGCATCTTATCCGAGTGG
TP53 R	TGGATGGTGGTACAGTCAGAGC
siNC-FAM	UUCUCCGAAGGUGUCACGUTT
siNC	UUCUCCGAAGGUGUCACGUTT
siGAPDH	UGACCUCAACUACAUGGUUTT
siP53	GACUCCAGUGGUAAUCUACTT
Scrambled siRNA	AGUGUAACGCUACCAUGCUTT

[a]F, forward primer; R, reverse primer.
